# Vascularized Fibula Grafts for Reconstruction of Bone Defects after Resection of Bone Sarcomas

**DOI:** 10.1155/2010/524721

**Published:** 2010-05-13

**Authors:** Michael Mørk Petersen, Dorrit Hovgaard, Jens Jørgen Elberg, Catherine Rechnitzer, Søren Daugaard, Aida Muhic

**Affiliations:** ^1^Department of Orthopaedic Surgery, Copenhagen University Hospital, Rigshospitalet Blegdamsvej 9, 2100 Copenhagen, Denmark; ^2^Department of Plastic Surgery, Copenhagen University Hospital, Rigshospitalet Blegdamsvej 9, 2100 Copenhagen, Denmark; ^3^Department of Paediatrics, Copenhagen University Hospital, Rigshospitalet Blegdamsvej 9, 2100 Copenhagen, Denmark; ^4^Department of Pathology, Copenhagen University Hospital, Rigshospitalet Blegdamsvej 9, 2100 Copenhagen, Denmark; ^5^Department of Oncology, Copenhagen University Hospital, Rigshospitalet Blegdamsvej 9, 2100 Copenhagen, Denmark

## Abstract

We evaluated the results of limb-sparing surgery and reconstruction of bone defects with vascularized fibula grafts in 8 consecutive patients (mean age at operation 13.6 years (range 4.1–24.2 years), female/male = 6/2) with bone sarcomas (BS) (osteosarcoma/Ewing's sarcoma/chondrosarcoma= 4/3/1) operated on form 2000 to 2006. The bone defects reconstructed were proximal femoral diaphysis and epiphysis (*n* = 2), humeral diaphysis (*n* = 2), humeral proximal diaphysis and epiphysis (*n* = 1), femoral diaphysis (*n* = 1), ulnar diaphysis (*n* = 1), and tibial diaphysis (*n* = 1). One patient with Ewing's sarcoma had an early hip disarticulation, developed multiple metastases, and died 9 months after the operation. The remaining patients (*n* = 7) are all alive 50 months (range 26–75 months) after surgery. During the follow-up the following major complications were seen: 1-2 fractures (*n* = 4), pseudarthrosis (*n* = 2), and hip dislocation (*n* = 1). Limb-sparing surgery with reconstruction of bone defects using vascularized fibular grafts in BS cases is feasible with acceptable clinical results, but fractures should be expected in many patients.

## 1. Introduction

Bone sarcomas (BSs) are rare but are most often highly malignant. In children and young adults, the most frequent histological types are osteosarcoma and Ewing's sarcoma, and the treatment of these bone sarcomas in the extremities consists of a combination of chemotherapy and surgical tumour resection [1, 2]. This combined treatment has led to a significantly increased survival since it was introduced in the seventies [3]. The five-year survival rate in patients with nonmetastatic disease is now above 70% [4, 5]. The introduction of chemotherapy combined with the developments of the last decades regarding imaging and surgical techniques including orthopaedic implant technology has made it possible to perform bone tumour resections as limb-sparing surgery in more than 80% of all cases, and without increased mortality [6, 7]. When a limb sparing tumour resection has been performed, various principles for reconstruction of the excised joint and adjacent bone exist: endoprosthetic replacement, vascularized bone graft, nonvascularized bone grafts (autograft or allograft, allograft combined with a vascularized bone graft, reimplantation of sterilised autologous bone), and bone distraction osteogenesis [2]. Reconstruction of joint and bone defects with an endoprosthetic replacement using special tumour prostheses is the preferred technique for reconstruction after resection of tumours of the extremities in adults, and with this technique limb salvage can be maintained in more than 80% of patients 20 years after the primary reconstruction [8]. Depending on different parameters such as the anatomical location of the tumour and patient age, the different techniques for reconstruction of joint and bone defects have their advantages and disadvantages. We present our results of reconstruction using vascularized fibula grafts after tumour resection in BS cases considered unsuitable for tumour prostheses because of very young age of the patients or an isolated diaphyseal tumour location of a long bone.

## 2. Patients and Methods

From 2000 to 2006, 8 consecutive patients (mean age at operation 13.6 years (range 4.1–24.2 years), female/male = 6/2) (Table 1) had limb-sparing surgery for BS at the Copenhagen University Hospital, Rigshospitalet, combined with a primary (*n* = 7) or secondary (*n* = 1) reconstruction of bone and/or joint defects using vascularized fibula grafts. The patients selected for this treatment regimen were cases considered unsuitable for reconstruction of the bone defects using tumour prostheses, because of very young age of the patients or the location of the tumour.

The distribution of the histological types of the BS included in the study was osteosarcoma (*n* = 4), Ewing`s sarcoma (*n* = 3), and chondrosarcoma (*n* = 1). Surgical resection of the BS resulted in bone defects of the femoral diaphysis including the proximal epiphysis (*n* = 2), the humeral diaphysis (*n* = 2), the humeral diaphysis including the proximal epiphysis (*n* = 1), the femoral diaphysis (*n* = 1), the ulnar diaphysis (*n* = 1), and the tibial diaphysis (*n* = 1). The average length of the resected bone was 15.7 (6–20) cm and the surgical margins obtained was wide (*n* = 6), marginal (*n* = 1), or intralesional (*n* = 1) (Table 1). 

Seven patients received preoperative chemotherapy, and 6 patients also had postoperative chemotherapy. Based on the evaluation of tumour necrosis done by the pathologist, the response to chemotherapy was classified as good with more than 90% tumour necrosis in 3 patients, moderate with 75% necrosis in one, poor with less than 50% necrosis in one, or not evaluable in 2 patients. The patient with intralesional margin also had a poor response to chemotherapy and had therefore external beam radiation therapy with a total dose of 54 Gy (Table 1).

**Figure 1:**
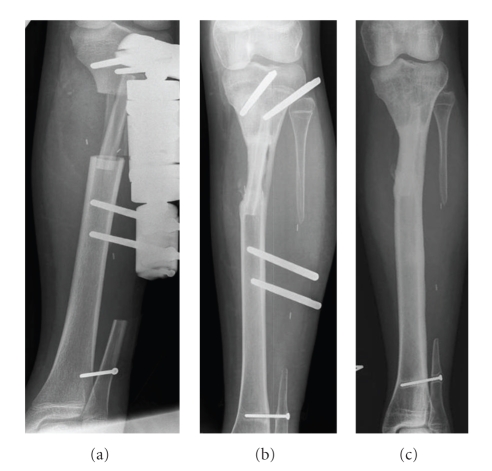
Resection of the proximal part of the tibial diaphysis performed because of an osteosarcoma and reconstruction of the bone defect with a double-barrelled pedicle vascularized fibula graft (patient 7). X-rays taken 9 days postoperatively (a), after 16.5 months when weight bearing without external fixation (fixation pins left until the effect of weight bearing was evaluated) had been started 3 weeks earlier (b), and status 53 months postoperatively (c).

With the exception of one patient who had tumour resection performed of the ulnar shaft and then 3 weeks later had reconstruction of the bone defect by a free vascuralized graft, all patients had tumour resection and reconstruction performed as a one-stage procedure with the tumour resections performed by orthopaedic surgeons, while plastic surgeons at the same time harvested the graft. Nine grafts were harvested (one patient had the femoral shaft reconstructed by two grafts) and with the exception of one graft used as a double barrelled pedicle-vascularized graft for reconstruction of an ipsilateral 6 cm tibial shaft bone defect (Figure 1), all grafts were free vascularized grafts. Three grafts included the fibular head including the joint surface with cartilage and the growth plate. While harvesting the grafts including the fibular head a piece of the anterior tibial artery including all branches to the proximal fibula was harvested and a reanastomosis of the anterior tibial artery was performed. The average length of the harvested grafts was 20.1 cm (15.5–24 cm) and four grafts also included a skin island. The average operation time was 8.1 hours (5.3–13.5 hours) with an average time of graft ischemia of 129 minutes (74–175 minutes). The grafts were fixed to the host bone by external fixation in 7 patients (in two of these patients combined with a plate or a single screw), and in one patient internal fixation with a plate was performed (Table 2). At the donor leg transfixation of the distal syndesmosis with a screw was performed in 4 patients (number 2, 4, 6, and 7) and fixation of the lateral collateral ligament of the knee was done with a staple in the 3 patients, who had a graft including the head of the fibula.

In the early postoperative phase the perfusion of the grafts was evaluated by laser Doppler flowmetry or Duplex. During the follow-up at various times most patients (*n* = 6) had bone scintigraphy for evaluation of the graft perfusion, local recurrence, and bone metastases. Bone healing and graft remodelling was evaluated by repeated plain X-rays, and with the exception of a patient suffering from a low-grade chondrosarcoma, all patients had regular chest-X-rays or CT scans of the lungs. From the patient files and a clinical examination, information regarding complications following surgery and clinical results assessed as the Musculoskeletal Tumour Society (MSTS) score [9] was recorded. In March 2009 we checked in a Danish nationwide register based on social security numbers (CPR-registry), if the individual patients were dead or still alive. Statistics: all results are given as mean and total range.

## 3. Results

One young patient aged 4 years suffering from Ewing's sarcoma had an early amputation (hip disarticulation) performed because of poor effect of chemotherapy with only marginal tumour resection 1.5 months after the primary operation with tumour resection and reconstruction of the proximal femur. He later developed multiple metastases and died of his disease 9 months after the operation. The remaining patients (*n* = 7) are all alive 60 months (33–102 months) after the date of diagnosis, and with the exception of one patient, who had a lung metastasis removed surgically 19 months after tumour resection, they were all (*n* = 7) without any signs of local recurrence or distant metastases during the clinical follow-up at 50 months (26–75 months) (Tables 1 and 3). 

**Figure 2:**
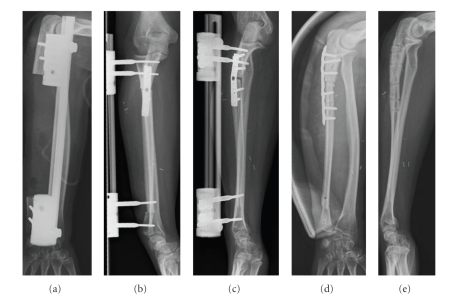
Status after resection of the ulnar diaphysis (patient 1) because of a low grade osteosarcoma (a), and reconstruction with a vascularized fibula graft 3 weeks later (b). A pseudathrosis developed at the proximal junction (c), and it was treated by bone grafting and plate osteosynthesis (d). The final result 2 years after reconstruction (e).

Bony union defined as a solid callus bridge between the fibula graft (both ends if reconstruction of a diaphyseal defect) and the host bone seen on plain X-rays was achieved after 15 (2–52) months (Table 3). When bone healing was obtained, the patients were allowed free use of the extremity including full weight-bearing of lower extremities. One of the patients developed a pseudarthrosis between the graft and the proximal ulna requiring bone grafting and a compression plate reosteosynthesis 9 months after the primary operation, and bony union was obtained 7 months later (Figure 2). Another patient, suspected from a bone scintigraphic examination 1 year postoperatively to have avascularity of at least one of the two grafts used for reconstruction of a femoral diaphyseal defect, had for several years no signs of bony healing at any of the bone graft ends and no remodelling of the graft. This patient had very long-term external fixation. Osteotomy of the grafts combined with bone grafting and later again bone grafting was performed twice until finally healing was obtained 52 months after the primary operation. Since almost no remodelling had occurred, hyperbaric Oxygen therapy was given following a stress fracture and again after removal of external fixation 67 months after the primary operation, and finally a good remodelling of the grafts was seen (Table 3).

We saw no complications in the immediate postoperative period and especially no problems with thrombosis of the fibular blood supply during or shortly after the operation. One patient had an infection at the donor site, but no graft infections were seen. Four patients had a total of 6 fractures (1-2 fractures each) and one of them could be classified as a stress fracture. With the exception of one fracture operated on with plate fixation all were treated conservatively without surgery. One of the fractures resulted in pseudarthrosis and therefore bone grafting and plate osteosythesis was performed later. As a consequence of a fracture of the proximal fibula (femur) that healed in an unfavourable position on conservative treatment, the hip joint became unstable with a tendency to dislocate, and therefore an osteotomy was performed (Figure 4). Furthermore two patients had one and two hammertoe operations, respectively, and one patient had a slight valgus deformity of the ankle joint of the donor leg (Table 3).

**Figure 3:**
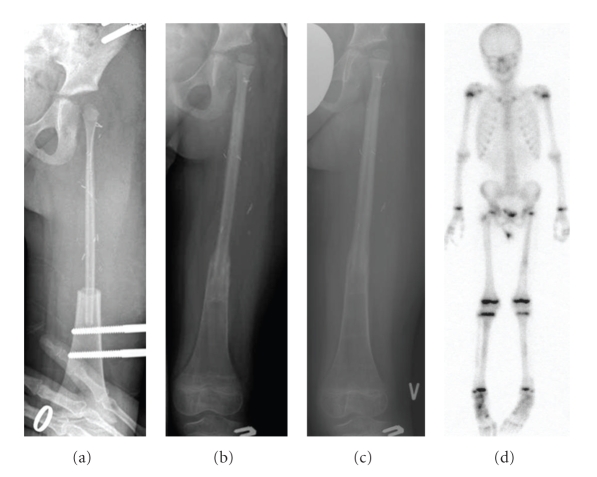
Resection of the proximal part of the femur because of Ewing's sarcoma and reconstruction with a vascularized fibula graft including the proximal epiphysis (patient 2). X-rays taken 2 weeks postoperatively (a), with follow-up after 7 months when weight-bearing was started (b), after 12 months when full weight-bearing had been allowed for 3 months (c), and finally a bone scan performed 12 months postoperatively (d).

**Figure 4:**
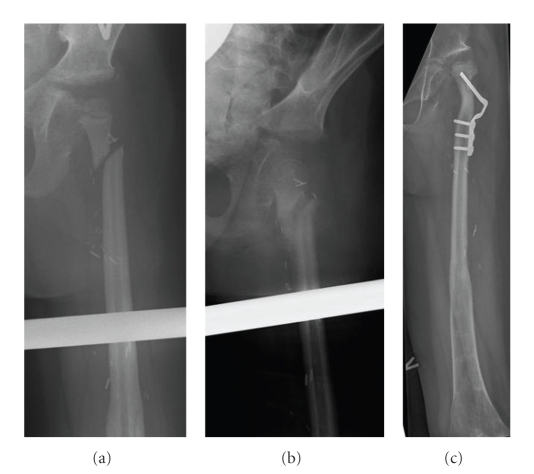
Resection of the proximal part of the femur because of Ewing's sarcoma and reconstruction with a vascularized fibula graft including the proximal epiphysis (patient 2). X-rays taken 13.5 months postoperatively after the patient had sustained a fracture from falling while doing gymnastics at school (a), and with follow-up 2 months after the fracture (b). X-ray 3 years after insertion of the fibula graft; a proximal osteotomy has been performed because the fracture healed in an unfavourable position leading to an unstable hip joint (c).

The average total MSTS score at the last clinical follow-up was 24 (18–29). One of the two patients with a low MSTS score of 18 was a girl with the femoral diaphysis and epiphysis replaced who suffered two fractures and a hip dislocation (Figures 3 and 4), and the other one was a girl with the humeral diaphysis replaced and additional postoperative external radiation therapy suffering from a fracture followed by a pseudarthrosis (Table 3). The average scores of the factors common to all the patients (*n* = 7) were pain 5 (5-5), function 3.7 (3–5), and emotional acceptance 3.4 (1–5). The average scores of the factors specific to upper extremity reconstruction (*n* = 4) were hand position 4.5 (3–5), manual dexterity 5 (5-5), and lifting ability 3.8 (2–5). The average scores of the factors specific to lower extremity reconstruction (*n* = 3) were supports 3.3 (1–5), walking ability 4 (3–5), and gait 3 (2–4).

## 4. Discussion

We report on 8 consecutive cases of children and young adults operated on for BS with limb-sparing tumour resection and reconstruction with vascularized fibular grafts. One patient who later died of his disease had an early hip disarticulation, while the remaining patients were alive on average 60 months (33–102 months) after the date of diagnosis, all with the fibula graft in situ and an average total MSTS score of 24 (18–29).

Only relatively few studies [10–18] evaluating the effect of reconstruction of bone defects with vascularized fibula grafts after operation for BS have been published previously, but because BS is a rare disease and the use of vascularized fibula grafts for reconstruction is a procedure only performed relatively seldom even in large centres, the studies are very inhomogeneous and not all of them have included patients suffering from BS exclusively. Other studies [19–23] have presented results after reconstruction of bone defects in BS patients with a combination of an allograft and a vascularized fibula graft.

In the present study bony union between the fibula graft and the host bone was achieved after a mean of 15 months (2–52 months). In previous studies [10–15] the average time until bone healing was achieved was very different, ranging from 4 to12 months after insertion of the fibula graft, and upper extremities seemed to heal faster than lower extremities [15]. In most studies nonunion occurs in 10–15% of cases [10, 12, 16]. In some studies, however, patients with nonunion or pseudarthrosis were excluded in the calculation of time to bony union and furthermore some had a high number of patients that were lost to follow-up [11, 12, 15]. Included in the figures of the present study are one patient who developed a pseudarthrosis between the graft and the proximal ulna requiring bone grafting and a compression plate reosteosynthesis until bony union was obtained (Figure 2), and another patient suspected to have avascularity of at least one of the two grafts used for reconstruction of a femoral diaphyseal defect, that did not achieve healing until 52 months after the primary operation. 

In the present study the most frequent major complication was fracture of the graft, which was seen in four patients, who had a total of 6 fractures. The number of fractures seen in previous studies is very variable, from no fractures [12, 15] to almost the same amount of fractures as in the present study [11, 14]. A frequent complication seen in 10%–30% of cases in most studies was deep infection [12, 14, 15], which did not occur in our material. However, the use of massive structural allografts or bridging plates could probably reduce the fracture rate, but the cost could be an increased risk of deep infections.

The functional results after reconstruction of bone defects with vascularized fibula grafts after operation for BS have only been evaluated very sparsely and only few of the studies include the exact figures of the MSTS score in the text [10, 13, 14]. We found an average MSTS score of 24 (80%), but in a large study by Germain et al. [13] presenting very briefly the results of 78 children, the MSTS score was surprisingly high with all average scores between 26.5 and 30. However, the MSTS score of the present study was comparable with previously published results by Rose et al. [14] and El-Gammal et al. [10], and furthermore the clinical results that can be obtained with reconstructions using a vascularized fibula graft is comparable to the long term results that can be obtained after endoprosthetic reconstruction after BS surgery with bone resections of the hip or knee joint [24, 25].

**Figure 5:**
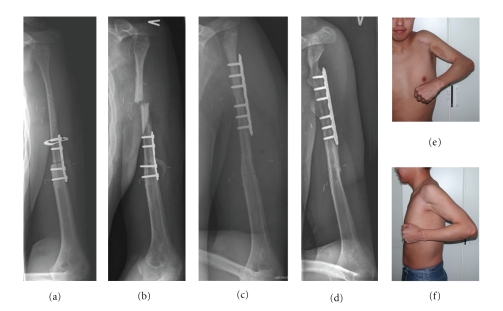
Resection of the proximal part of the humerus because of an osteosarcoma and reconstruction with a vascularized fibula graft including the proximal epiphysis (patient 5). X-rays taken 1 month postoperatively (a), and after 8 months when the junction was healed but the patient had just sustained a fracture of the graft (b) treated by plate ostesynthesis (c). 29 months after insertion of the graft, where the patient has had one more fracture just above the plate healed on conservative treatment; X-ray (d) and clinical photos showing active abduction (e) and extension (f) of the shoulder joint just before removal of the plate.

The use of the fibular head including the joint cartilage and the growth plate in vascularized fibula graft transfer for bone and joint defects after BS resection have mainly been done for reconstructions performed of the upper extremities [13, 14, 16–18]. We performed one reconstruction of the proximal humerus with the use of a vascularized fibula graft with the fibular head including joint cartilage and growth plate in a 15-year-old boy, and in spite of two fractures and limited function of the shoulder joint the patient had a pain free well functioning upper extremity (Figure 5). 

The first study describing a case using a fibula graft including the proximal joint cartilage and the growth plate for reconstruction of the proximal femur and hip joint in a BS patient was published by Manfrini et al. [19] in 2003. They performed a reconstruction of the left proximal femur after a 13 cm bone resection for Ewing's sarcoma in a 4-year-old girl. The vascularized fibula graft was inserted in and protected by a massive proximal femur allograft. To our knowledge, the study by Germain et al. [13] is the only other study that has presented a case where a fibula graft including the proximal joint cartilage and the growth plate was used for reconstruction of the proximal femur and hip joint in a BS patient. The study presented very briefly the results of 78 children operated on with resection of a BS and reconstruction with a vascularized fibula graft during a period of 15 years. In four cases reconstruction of the proximal femur or humerus was performed with a graft including the growth plate and epiphysis. The article does not give precise information regarding the distribution of lower extremity and upper extremity reconstructions, but from the serial X-rays shown, we can see that at least one of the four cases was a lower extremity reconstruction. We performed two reconstructions using a vascularized fibula graft including the growth plate and epiphysis for reconstruction of the proximal femur in two children aged, respectively, 4 and 6 years. One of the patients later died of his disease and also had an early hip disarticulation, performed because of poor effect of chemotherapy and only marginal tumour resection. The other patient was free of disease and had a pain free acceptable limb function even though she had sustained two fractures and a hip dislocation (Figures 3 and 4). The alternative surgical treatment to these two cases would have been an amputation (hip disarticulation) or perhaps a type-B-IIIa hip rotationplasty [26].

## 5. Conclusion

Reconstruction using vascularized fibula grafts after tumour resection in BS cases considered unsuitable for tumour prosthesis because of very young age of the patients or an isolated diaphyseal tumour location of a long bone is feasible, but complications, especially fractures, should be expected in many patients.

## Figures and Tables

**Table 1:** Patient and tumour data.

Patient	Location	Age at operation [years]	Sex	Histological type	Chemo-therapy	Postoperative radiation therapy	Surgical margin	Tumor necrosis	Local recurrence	Metastases (during follow-up)	Alive
1	Ulnar diaphysis	21.1	F	Osteosarcoma (low malignant)	Preoperative only (Cisplatin + Adriamycin)	No	Wide	Could not be evaluated	No	No	Yes
2	Femoral diaphysis and proximal epiphysis	6.9	F	Ewing's sarcoma	Pre- and postoperative (Euro-ewing99)	No	Wide	100%	No	No	Yes
3	Humeral diaphysis	12	F	Ewing's sarcoma	Pre- and postoperative (Euro-ewing99)	Yes (54 GY)	Intralesional	10%	No	No	Yes
4	Humeral diaphysis	12.4	M	Chondrosarcom (low malignant)	No	No	Wide	Not evaluated (irrelevant)	No	No	Yes
5	Humeral diaphysis and proximal epiphysis	15.7	M	Chondroblastic osteosarcoma	Pre- and postoperative (SSG14)	No	Wide	75%	No	No	Yes
6	Femoral diaphysis and proximal epiphysis	4.1	F	Ewing's sarcoma	Pre- and postoperative (Euro-ewing99)	No	Marginal	30%	No	Yes Multiple	No
7	Tibial diaphysis	12.2	F	Osteosarcoma	Pre- and postoperative (SSG14)	No	Wide	100%	No	No	Yes
8	Femoral diaphysis	24.2	F	Osteosarcoma	Pre- and postoperative (Cisplatin + Adriamycin)	No	Wide	99%	No	Yes Lung (radical surgery)	Yes

F = female, M = male.

**Table 2:** Operative data.

Patient	Location	Bone defect length [cm]	Fibula length [cm]	Skin island	Operation time^a^ [hours]	Graft ischemia time [minutes]	Donor vessels	Initial graft fixation
1	Ulnar diaphysis	20	20	Yes	5.3 (only reconstruction)	136	Peronea	External fixation and internal (a bridge plate)
2	Femoral diaphysis and proximal epiphysis	19	19 (fibular head included)	No	13.5	160	Peronea +anterior tibial	External fixation
3	Humeral diaphysis	19	22	No	7.0	110	Peronea	External fixation
4	Humeral diaphysis	13.5	18	No	7.9	113	Peronea	External fixation and internal (a single screw)
5	Humeral diaphysis and proximal epiphysis	16	24 (fibular head included)	Yes	7.2	102	Peronea + anterior tibial	Internal fixation (a bridge plate)
6	Femoral diaphysis and proximal epiphysis	13	15.5 (fibular head included)	No	10.5	74	Peronea + anterior tibial	External fixation
7	Tibial diaphysis	6	18	Yes	5.5	160	Peronea (Pedicle graft)	External fixation
8	Femoral diaphysis	19	22 (left) 22 (right)	Yes No	8.1	130 175	Peronea	External fixation

^a^Tumor resection and reconstruction.

**Table 3:** Results.

Patient	Location	Follow-up Survival after diagnosis [months]	Follow-up Clinical after surgery [months]	Enneking score	Bone healing [months]	Fractures (and treatment)	Other surgical complications and treatment
1	Ulnar diaphysis	35	26	27	16	No	Operation for a nonunion Hammertoe operation Removal of plate (after union)
2	Femoral diaphysis and proximal epiphysis	43	37	18	4	Two (both treated conservatively)	Hip dislocation (osteotomy of the proximal part of the graft)
3	Humeral diaphysis	62	55	18	10	One (treated conservatively and resulted in pseudarthrosis)	Pseudarthrtosis after a fracture (treated by bone grafting and plate osteosythesis)
4	Humeral diaphysis	102	72	29	2	No	Two hammertoe operations
5	Humeral diaphysis and proximal epiphysis	33	29	27	8	Two (plate osteosythesis and conservative treatment)	Infection at donor site
6	Femoral diaphysis and proximal epiphysis	13	9	—	—	No	Hip disarticulation (poor response to chemotherapy and only marginal resection)
7	Tibial diaphysis	63	55	29	16	No	Slight valgus deformity of donor ankle
8	Femoral diaphysis	83	75	21	52	One (stress fracture while still using external fixation—treated conservatively)	Slow healing and remodeling of the grafts: 1. Osteotomy and bone grafting, 2. Bone grafting x 2 2. Long term external fixation 3. Hyperbaric Oxygen therapy
